# Screening for financial vulnerability in a paediatric hospital setting: a pilot study of the Financial Risk Questionnaire (FRIQuest)

**DOI:** 10.1007/s00431-026-07474-5

**Published:** 2026-10-12

**Authors:** Ashley J. P. Smit, Bente Q. Alderlieste, Lequisha G. Suisse, Ingrid A. Peters, Nicole Lucassen, Koen F. M. Joosten

**Affiliations:** 1 Department of Neonatal and Paediatric Intensive Care, Erasmus MC - Sophia Children’s Hospital, University Medical Center Rotterdam, Dr. Molewaterplein 40, Rotterdam, 3015 GD Netherlands; 2 Department of Quality and Patient Care, Erasmus MC, University Medical Center Rotterdam, Rotterdam, Netherlands; 3 Department of Psychology, Education and Child Studies, Erasmus University Rotterdam, Rotterdam, the Netherlands

**Keywords:** Poverty, Financial vulnerability, Child health, Paediatricians, Screening, Questionnaire, Digital, Implementation

## Abstract

Growing up in poverty can significantly influence child health and access to healthcare. Paediatric healthcare providers can play an important role in identifying families experiencing financial vulnerability. However, various barriers prevent standard poverty screening in paediatric care. To support this, we developed a digital financial vulnerability screening questionnaire: the Financial Risk Questionnaire (FRIQuest). This study describes its development and evaluates its implementation, perceived usefulness, acceptability, and user friendliness. This mixed-methods implementation study was conducted in a tertiary paediatric hospital in Rotterdam, the Netherlands. Quantitative data included response rates, FRIQuest item responses, parent evaluation survey data, and healthcare provider compliance rates. Qualitative data were obtained through interviews with parents and paediatric healthcare providers. The FRIQuest was sent out to parents of 1206 unique patients. A total of 485 (40.2%) completed the FRIQuest, of whom 22.0% reported (sometimes) experiencing financial vulnerability. Parents generally found the questionnaire easy to complete and felt comfortable answering its questions. They considered it appropriate for healthcare providers to address financial vulnerability, preferably as part of a broader conversation about family wellbeing rather than as a stand-alone topic. Healthcare providers reported that the questionnaire was useful for identifying families in need and was feasible in clinical practice. However, various barriers affected response rates, healthcare compliance, and initiation of conversations.

*Conclusion*: FRIQuest is a feasible, generally acceptable, and potentially useful digital questionnaire to support the identification of financial vulnerability in paediatric hospital care. This study highlights that successful implementation requires not only suitable questionnaires, but also inclusive implementation strategies, provider engagement and support, and access to social support. Future research should evaluate its impact on referrals to support services and socioeconomic inequalities in child health.

**Table:** 

**What is Known:** • *Growing up in poverty is associated with poorer health and reduced access to healthcare.* • *Paediatricians can contribute to improving child health outcomes by addressing financial vulnerability within clinical care. However, various barriers prevent standard financial vulnerability screening.*	
**What is New:** • *The Financial Risk Questionnaire is a feasible and generally acceptable digital questionnaire to support identification of financial vulnerability in paediatric hospital care.* • *Besides suitable questionnaires, successful financial vulnerability screening requires inclusive implementation strategies, provider support, and organisational embedding.*

**Supplementary Information:**

The online version contains supplementary material available at https://doi.org/10.1007/s00431-026-07474-5.

## Introduction

Growing up in poverty and the accompanying stress has been associated with increased paediatric morbidity [1–3]. For example, children from a disadvantaged socioeconomic background have significantly higher odds of being diagnosed with a chronic illness than their more affluent peers. The most pronounced disparities are observed in conditions such as psychological disorders, intellectual disability, and asthma [4]. Beyond morbidity, the impact of poverty is visible in lifestyle behaviours and in access to and use of healthcare services [5, 6]. While poverty is commonly defined through income thresholds internationally, material deprivation and financial vulnerability can occur independently of income level and influence wellbeing and healthcare use in similar ways [7–10]. Therefore, in this paper, the term financial vulnerability is used to refer to the broader experience of poverty, irrespective of income level.

While addressing financial vulnerability requires structural and policy-level solutions, paediatric healthcare providers may play an important role in recognising signs of financial vulnerability and refer families to support services. Garg and colleagues showed that referral rates to support services increased from just 8% to 70% when structured screening for social determinants was implemented [11]. Though direct evidence on child health outcomes remains limited, identification and referral of parents in poverty are associated with reductions in unmet social needs, emphasising the short-term benefits of such practices while suggesting potential for longer-term improvements in child health [12]. Despite this evidence, financial vulnerability screening has not yet been integrated into Dutch standard paediatric care. A previously conducted survey showed that 84% of Dutch paediatricians considered it important to play a role in identifying and discussing financial vulnerability with their patients, consider affordable solutions, and refer families to appropriate support services [13]. However, multiple competing priorities and barriers prevent this from occurring in practice, including consultation time constraints, uncertainty about the appropriateness of addressing poverty within clinical care settings, feeling under-skilled and uncomfortable addressing the topic, and a lack of (knowledge about) available support resources [13]. Accordingly, Purkey et al. reported that general practitioners used a verbal screening question to assess families’ experiences of poverty in only 9% of consultations, highlighting the ongoing barriers healthcare providers encounter when attempting to initiate conversations about poverty with families [14].

Screening questionnaires for patients could be a solution to overcome some of these barriers, by providing a structured and less confrontational means of initiating conversations about financial vulnerability [15]. Zheng and colleagues showed the feasibility and acceptability of such a questionnaire among patients and healthcare providers, reporting a 92% response rate and a 44% incidence of household material hardship (the inability to afford or access essential goods and services) [16]. Their approach relied on the Psychosocial Assessment Tool within a paediatric oncology referral centre. However, this instrument was considered less fitting for our context, which involves all paediatric specialities and a need for a broader focus on poverty, involving subjective financial strain and material deprivation.

### The current study

To address this gap, we developed and piloted the Financial Risk Questionnaire (FRIQuest), a digital screening questionnaire to facilitate the identification of financial vulnerability among parents of paediatric patients. This study outlines the development, implementation and evaluation of the FRIQuest in a paediatric hospital setting. Our primary research question was as follows: “Does the FRIQuest help paediatric healthcare providers to identify and facilitate discussion of financial vulnerability among parents of their patients?”. Our secondary research questions aim to assess the implementation, the acceptability and user friendliness of FRIQuest among parents and paediatric healthcare providers.

## Methods

### Study design and setting

We conducted a mixed-methods implementation study that developed, implemented, and evaluated the FRIQuest in routine clinical care in a paediatric tertiary hospital in Rotterdam, the Netherlands. As Rotterdam has the second highest child poverty rate in the country, the setting was particularly fitting for piloting the FRIQuest. Four paediatric departments voluntarily participated in the pilot study. Data were collected from January 2025 to April 2026. The mixed-methods study design allowed for a broad evaluation of the implementation of FRIQuest by combining quantitative outcomes with qualitative insights. Quantitative data (including response rates, responses to individual FRIQuest items, parent evaluation survey responses and healthcare compliance rates) were collected continuously during the pilot period to assess uptake, completion, and parental acceptability and user friendliness. The Checklist for Reporting Results of Internet E-Surveys was followed in reporting on the evaluation survey component of this study [17]. Qualitative interviews with parents and healthcare providers were conducted during the pilot period to explain and contextualise quantitative findings. The qualitative component was reported in accordance with the consolidated criteria for reporting qualitative research (COREQ) checklist (see Supplementary file 1) [18].

### Development of the FRIQuest

The FRIQuest (Supplementary file 2) was developed using the Social Needs Screening Toolkit and the Psychology Inventory of Financial Scarcity as foundational resources [19, 20], as these focus on material hardship in healthcare contexts and subjective experiences of poverty, respectively. This aligns with the study’s goals of focusing on financial vulnerability, rather than income poverty, as focusing solely on income may overlook families whose financial strain still affects health, healthcare access, and use [8–10]. Paediatric healthcare providers and experts in financial vulnerability contributed to the development of the questionnaire to ensure relevance for clinical practice.

The FRIQuest comprised seven items: the first question assessed general financial concerns, followed by four questions assessing whether there was enough money for fixed expenses, healthy food, participation in sports, and medical care. These were selected based on their association with child health, and perceived clinical relevance by stakeholders. The final two questions asked whether respondents wished to receive (more) assistance for financial concerns and inquired if respondents wished to provide any additional comments on the topic with an open-ended response.

The FRIQuest was written at B1 language level to enhance comprehensibility and used three response options as recommended in guidelines on comprehensible questionnaires [21]. The FRIQuest was available in Dutch and English.

### Implementation of the FRIQuest

#### Integration of the FRIQuest in existing structures

The FRIQuest was embedded within the hospital’s digital value-based health care (VBHC) questionnaire infrastructure [22]. VBHC aims to maximise value for patients, primarily measured with patient-reported outcome measures (PROMs). A prerequisite for implementation of the FRIQuest was that departments also distributed the Generic PROM Child, which is used in the VBHC program to assess general quality-of-life domains. This approach intended to position financial vulnerability alongside other quality-of-life domains to normalise discussion of the topic within clinical consultations.

Questionnaire distribution was automated based on appointment codes recorded in the electronic patient record, enabling departments to tailor eligibility (e.g. new patients vs. control patients). In addition, the frequency of distribution could be adjusted, although it was most often distributed annually. One department additionally filtered on zip code to target Rotterdam-based patients only.

Parents received an automated email invitation 1 week prior to their child’s appointment, containing a link to the patient portal where they could complete the FRIQuest. An introduction text explained the purpose of the questionnaire, stating why financial vulnerability is relevant to health and healthcare (Supplementary file 2). A reminder was sent 3 days before the appointment if the questionnaire had not yet been completed.

**Fig. 1:**
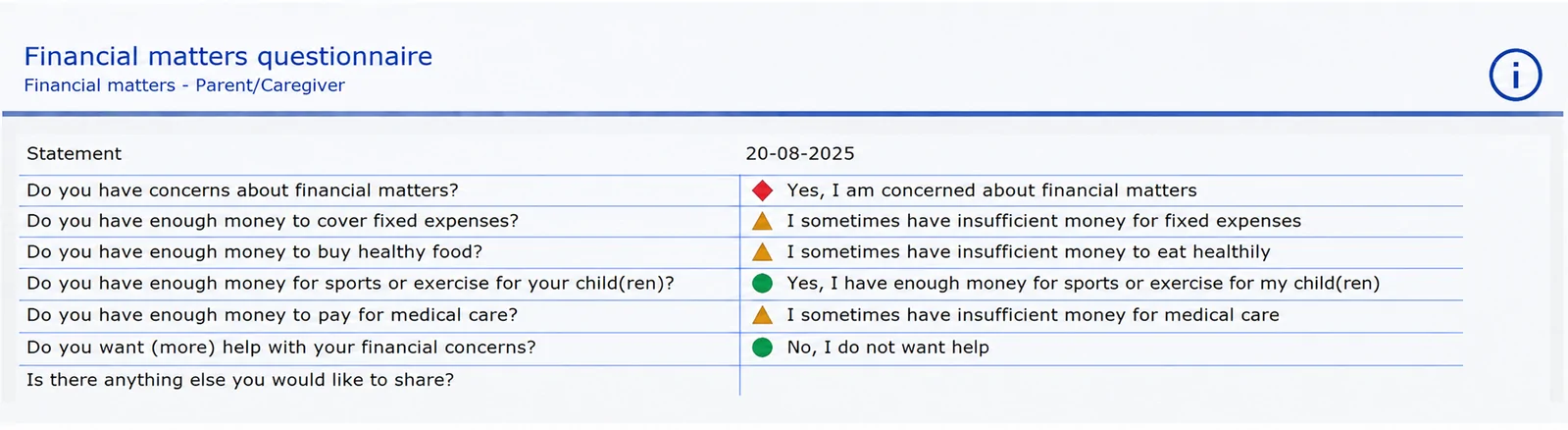
FRIQuest results displayed in the VBHC dashboard

Healthcare providers could access responses via the VBHC dashboard within the electronic patient record. The dashboard displayed the individual questions of the FRIQuest and given answers by parents. A colour-coded indicator summarised responses to the FRIQuest: red (financial vulnerability), orange (some financial vulnerability), and green (no financial vulnerability; see Fig. 1).

#### Implementation approach

Department participation in piloting FRIQuest was voluntary. A multidisciplinary team with expertise in financial vulnerability, quality of care, implementing digital questionnaires, and healthcare delivery supported implementation among departments. Activities included department outreach, presentations, IT configuration and healthcare provider kick-off trainings on dashboard use, how to discuss the FRIQuest results with parents, and referral pathways. Implementation was refined iteratively based on feedback from healthcare providers and families, allowing for timely adjustments to improve integration and engagement of FRIQuest.

#### Referral pathways

Referral pathways were developed to facilitate healthcare providers to take appropriate action when identifying financial vulnerability and to ensure families received the support they needed. Referrals could be initiated based on FRIQuest results as well as healthcare providers’ observations during consultations.

For Rotterdam-based families experiencing financial vulnerability, a pilot collaboration with the municipality of Rotterdam was established. Healthcare providers could either schedule an appointment for their patients through a Microsoft Booking system or provide families with a leaflet containing the consultant’s contact details and a QR code linking to the booking system, allowing families to arrange an appointment themselves. The implementation and experiences of this pilot collaboration will be described in a separate article. Families residing outside Rotterdam were referred to the hospital’s patient service centre, which could assist with identifying and contacting appropriate local support services. Medical social workers remained available to support families with broader psychosocial needs.

### Inclusion criteria, recruitment, and study sample

Both parents of patients and healthcare providers were asked to evaluate the FRIQuest. There were no formal inclusion or exclusion criteria for participation in the evaluation, other than the requirement that participants were 18 years of age or older and had either received (parents) or worked with (healthcare providers) the FRIQuest. The parents received an invitation to the evaluation questionnaire about the FRIQuest by e-mail, 1 week after their child’s outpatient appointment. Those who indicated that they completed the FRIQuest got the question whether they were willing to participate in an interview. Parents who provided contact details were then purposively approached by phone for participation in this interview. As the interviews were intended to provide contextual understanding and support interpretation of the quantitative findings, recruitment was guided by the inclusion of diverse perspectives rather than the achievement of data saturation. The participating healthcare providers of the four departments were approached by email to participate in an interview six months after FRIQuest implementation. There were no other people present besides the researcher(s) and the interview participant(s).

Over a 15-month period, 1206 invitations for the post-visit evaluation questionnaire (Supplementary file 3) were sent to parents; 151 responses (12.5%) were received of which 112 gave permission for the use of their answers for research. Their characteristics are described in Supplementary file 4. Twenty-one respondents consented to contact for follow-up research; six were approached and four completed an in-depth interview (two reported no financial vulnerability; one reported prior financial vulnerability but no current concerns due to financial administration; one reported ongoing vulnerability). Reasons for non-participation were not registered. Four paediatricians and one nurse practitioner participated in four interviews. All healthcare providers were female.

### Outcome measures

Study outcomes were structured around three evaluation domains: (1) implementation outcomes, (2) usefulness of the FRIQuest as a digital screening questionnaire, and (3) acceptability and user friendliness.

Implementation success was assessed by the number of families completing the FRIQuest and healthcare professional compliance (defined as the number of times they opened their patients’ completed FRIQuests in the VBHC dashboard). Other implementation outcomes included barriers and facilitators to completion and compliance identified in the interviews. Usefulness of FRIQuest as a screening questionnaire and conversation starter was assessed by the number of families identified by the FRIQuest as experiencing financial vulnerability, and by patients’ and healthcare providers’ perceptions of its value in identifying and discussing financial vulnerability. Actual discussion rates were not systematically recorded during the implementation period. Acceptability and user friendliness were assessed using items from the evaluation questionnaire and parent interviews that examined perceived comfort and ease of use.

### Data collection and analysis

#### Quantitative data collection and analysis

Quantitative data were derived from responses to the FRIQuest (e.g. number of questionnaires in which financial vulnerability was reported), from routine metadata generated through the value-based health care (VBHC) infrastructure (e.g. number of questionnaires sent and completed, and healthcare compliance rates), and an evaluation survey on FRIQuest acceptability, user friendliness, and healthcare provider follow-up (Supplementary file 3). Analyses of questionnaire distribution and response rates were based on unique patients only; if a patient received or completed the questionnaire multiple times during the study period, they were counted once, and only the first completed questionnaire was retained for analysis.

The evaluation survey was developed and administered in LimeSurvey (Version 2.06). Responses on the evaluation questionnaire were exported to and analysed using Excel. The data were used to identify response rates and summary scores for the questions, analysed by AJPS. In addition, the characteristics of parents who completed the evaluation questionnaire were described. Only evaluation questionnaires for which at least the first question was answered, and respondents gave informed consent for the use of their data for this study, were included in the analysis (*n* = 112). Fifty-three (32.1%) entries were excluded.

Quantitative analyses consisted of descriptive statistics only. Frequencies, proportions, means, and medians were used as appropriate. These were calculated using Microsoft Excel version 2508 (Microsoft Corporation, Redmond, WA, USA).

#### Qualitative data collection and analysis

Semi-structured interviews were conducted by AJPS, BQA, and LGS, all trained in qualitative interviewing and not involved in the clinical care of participants, to minimise potential bias. Interviews followed predefined topic guides that were tested within the project team (see Appendices 5 and 6). Interviews with parents took place by telephone. Interviews with healthcare providers were in person at Erasmus Medical Center, online or by telephone. One healthcare provider was unable to attend the full interview due to a late arrival. Therefore, the interview was repeated to ensure complete data capture from all participants. The interviews lasted approximately 40 min and were audio-recorded with participants’ consent. Audio recordings were transcribed verbatim with an automated transcription software and checked for accuracy by AJPS, BQA, and LGS. No field notes were made during the interviews. Transcripts were not returned to participants for comments.

Interviews were analysed using directed qualitative content analysis [23]. Alternating, AJPS, BQA, and LGS independently coded transcripts based on the survey’s evaluation domains (usefulness, acceptability, and user friendliness). Codes were developed directly from the data to capture parents’ experiences and perceptions of the FRIQuest, such as ‘trust in the healthcare provider’, ‘feelings of surprise’, ‘ease of completion’, and ‘emotional responses to the questions’. Coding was supported by Atlas.ti, version (v9.26.0). The researchers met regularly to compare interpretations and refine codes. Discrepancies were resolved through discussion. A reflexivity of the interviewers and coders can be found in Supplementary file 7. Participants were not asked to provide feedback on the findings of this study.

## Results

### Implementation

This section presents the findings of the implementation outcomes, including parents’ completion of the FRIQuest, healthcare provider compliance, and reported follow-up discussions, complemented by findings from the survey and interviews.

#### FRIQuest completion and non-completion

Over the 15-month pilot period, the FRIQuest was sent out to parents of 1206 unique patients and completed 485 times (40.2%). 59.8% of parents did not complete the FRIQuest. Several reasons for non-completion were reported by 31 evaluation survey respondents. The most reported reason was unawareness of the questionnaire (*n* = 10, 32.3%), followed by not having a digital identification necessary to access the patient portal (*n* = 7, 22.6%), and noticing too late that a questionnaire needed to be completed (*n* = 6, 19.4%). The full scope of reasons can be found in Supplementary file 4. As explained by one healthcare provider, a key reason for non-response to the questionnaire could be related to limited digital skills and internet access, which she supposed to be increasingly prevalent in people with financial vulnerability:

“I think that perhaps the group you actually target, that they do not always complete that questionnaire. [...] Because they must complete it, [and they] need to have access to internet. And these often form a barrier.” Healthcare provider 3

The interviews with parents explored whether the relationship with the healthcare provider influenced completion of the questionnaire. One participant emphasised that trust in her healthcare provider contributed to her comfort in completing the questionnaire, while another participant indicated that she would be willing to disclose financial vulnerability even in a new hospital setting.

#### Healthcare provider compliance and follow-up discussion

Although most healthcare providers considered the FRIQuest an appropriate questionnaire for identifying financial vulnerability, its use during patient consultations varied considerably. Compliance of healthcare providers (the number of times they opened completed FRIQuests in the VBHC dashboard) varied from 4 to 78%. There are various reasons that could explain the relatively low compliance numbers. Multiple healthcare providers mentioned that they either lacked enough time in their preparation to examine the given answers or that they had simply forgotten to check the answers beforehand.

One healthcare provider emphasised that new instruments require time to become embedded in routine practice:

“We’re not used to it yet… we need to get into it a bit.” Healthcare provider 4.

She emphasised that regular updates and check-ins from the implementation team could serve as an important stimulus for routinely integrating the FRIQuest into practice.

Even when healthcare providers observed in their preparation that financial vulnerability was disclosed in the FRIQuest, they did not always follow-up on the topic with their patients. They mentioned that addressing financial strain does not always fit during the appointment, particularly when more pressing topics required attention and may already overwhelm patients, or when time constraints limit the scope of the consultation. For one healthcare provider, these barriers were especially present in consultations with patients who visit the hospital for the first time with their parents, highlighting that the relationship between the healthcare provider and parents of patients can play a role in discussing financial vulnerability:

“I must say that with new patients I still find it quite difficult… They come with a new problem, often from another hospital, they have invested all their hope in this, expecting that things will finally move forward, and then to start talking about financial problems... [...] But I find it more natural to talk about with people you have known longer, and whose home situation you are more familiar with, and for me, as healthcare provider, also more relevant.” Healthcare provider 4

The lack of discussing financial vulnerability was also reflected in the evaluation questionnaire and the interviews with parents, where multiple parents who disclosed financial vulnerability through FRIQuest reported receiving no follow-up from their child’s healthcare provider. However, as one parent explained, these concerns were sometimes already known and previously addressed by the healthcare provider, making further discussion unnecessary. Nevertheless, three healthcare providers shared that they have little difficulty discussing financial vulnerability reported in FRIQuest and described situations in which they had done so.

### Usefulness

This section presents the responses to the individual FRIQuest items and the (perceived) usefulness of FRIQuest as a screening questionnaire and conversation starter.

#### Responses to FRIQuest items

Of 485 completed questionnaires, 414 (85.4%) consented to the use of their data for research. Of these, 91 parents (22.0%) reported financial concerns, material hardship, or both. Specifically, 74 parents (17.9%) reported having some or substantial financial concerns, and 57 parents (13.8%) reported some or substantial material hardship, including insufficient financial resources for fixed expenses (*n* = 16, 3.9%), healthy food (*n* = 20, 4.8%), participation in sports or exercise (*n* = 38, 9.2%), and medical care (*n* = 30, 7.2%). Of these 91 parents, 40 (44.0%) reported both financial concerns and material hardship, and 7 respondents (7.7%) indicated interest in receiving assistance for their financial situation. Detailed results for each question can be found in Table 1.

**Table 1:** Distribution of parent responses to individual FRIQuest items (*N* = 414)

FRIQuest item	Answer (*n*, %)		
	Yes	Sometimes	No
1. Do you have concerns about financial matters?	8 (1.9%)	64 (15.5%)	342 (82.6%)
2. Do you have enough money to cover fixed expenses?	398 (96.1%)	9 (2.2%)	7 (1.7%)
3. Do you have enough money to buy healthy food?	394 (95.2%)	17 (4.1%)	3 (0.7%)
4. Do you have enough money for sports or exercise for your child(ren)?	376 (90.8%)	28 (6.8%)	10 (2.4%)
5. Do you have enough money to pay for medical care?	384 (92.8%)	24 (5.8%)	6 (1.4%)
	Yes	I already receive enough help	No
6. Do you want (more) help with your financial concerns?	7 (1.7%)	38 (9.2%)	369 (89.1%)
	Thematic categorization of answers (*n*)		
7. Is there anything else you would like to share?	• Comment or question about the goal of the FRIQuest (5)		
	• Comment on high travel and/or healthcare expenses (5)		
	• Further clarification of financial vulnerability (3)		
	• Confirm the absence of financial vulnerability (3)		
	• Expressing surprise or displeasure about receiving FRIQuest (2)		
	• Other (4)		

Twenty-one parents (5.1%) provided a response to the open-ended question in FRIQuest that asked if there was anything they wished to share. Their answers were thematically categorised and can be found in Table 1. Some parents clarified their financial vulnerability or reported high cost of travel or medical care. Within the interviews, participants also gave various examples of financial vulnerability, and how they impact access to and use of healthcare:

“If I had to go to [the hospital in] Rotterdam, then I [...] I would panic. Like yes, but how do I do that, because the public transport is already €50. Back then I had €60 per week [to spend], so then [we would have] only €10 left for the rest of the week to eat.” Parent 2 (previously experienced financial vulnerability)

“Because for a long time I didn’t really use it [the prescribed topical medication for my child]. I just went to buy petroleum jelly at the [drugstore], because yeah €10 [...] is a bit pricey.” Parent 4 (currently experiencing financial vulnerability)

#### Perceived usefulness

Two healthcare providers reported in their interviews that the FRIQuest helped uncover financial vulnerability in families they would not have anticipated otherwise, contributing to their perceived usefulness of the FRIQuest in identifying such concerns. In addition, the FRIQuest increased healthcare providers’ awareness of financial vulnerability and its possible impact on access to care for families:

“Definitely, awareness has become much greater now, absolutely… you pay more attention to those signals you might have picked up before, like if someone says, ‘I have to pay for this myself, I can’t come again,’ and so on.” Healthcare provider 3.

According to one healthcare provider, an additional advantage of using a questionnaire to assess financial vulnerability is that it creates an opportunity to initiate conversation about the topic:

“No, so it is actually very easy to discuss. It provides an opening. Of course, you could simply ask, ‘how are things financially?’ [...], but it is easier to discuss with such a questionnaire and it also ensures that the issue is explicitly included on the agenda of topics to discuss, because otherwise you often get caught up in all the medical aspects and developmental issues, and finances tend to receive far less attention in our consultations.” Healthcare provider 2

Another beneficial outcome of the FRIQuest, according to both healthcare providers and patients, was its role in normalising conversations about financial vulnerability and reducing stigma about the topic for parents of patients.

### Acceptability and user friendliness

This section summarises parents’ and healthcare providers’ reflections on the acceptability and user friendliness of FRIQuest, complemented by survey responses from parents. Findings on acceptability and user friendliness are presented across four key areas: the appropriateness of addressing financial vulnerability in paediatric care, comfort while completing FRIQuest, perceptions of the questionnaire as a method to enquire about financial vulnerability, and the ease of completing or engaging with FRIQuest.

#### Relevance and appropriateness of addressing financial vulnerability in paediatric care

Respondents to the survey were asked if they found it useful that their child’s healthcare provider knew about existing financial vulnerability. Most respondents answered that they find it useful (*n* = 28, 53.8%), some were neutral (*n* = 13, 25.0%), and others did not find it useful (*n* = 11, 21.2%). Among those who were neutral or did not find it useful, two respondents indicated in the open-ended responses that they did not understand why they were asked about their financial matters. In addition, two respondents indicated that they did not consider it the responsibility of a hospital to enquire about financial vulnerability. Four respondents explained that addressing financial vulnerability in paediatric care through FRIQuest was just not useful for them personally, because they did not experience financial vulnerability, but they felt like it could benefit others.

Nevertheless, five respondents explicitly praised the hospital’s awareness of financial vulnerability in their open-ended responses. Similarly, all interview participants emphasised that inquiring about financial vulnerability was fitting in a hospital setting, which was either immediately clear or became clearer during the interview:

“After you explained it, I understand it better, because at first I was like ‘yeah, but what does that [financial vulnerability] have to do with healthcare?’ But yes, if you basically don’t have the money to go to the appointment, then your child also doesn’t get the care that they need.” Parent 2 (previously experienced financial vulnerability)

However, addressing financial vulnerability felt appropriate as part of a general enquiry into how a family is doing, rather than as a separate stand-alone question:

“Yes, definitely. If you come to the hospital often, then not only the patient themselves is, so to speak, brought into focus, but really the family as well, right? Because where does such a patient live? What things are going on there? Yes, and I think financial concerns can definitely be part of that.” Parent 1 (no financial vulnerability)

Overall, healthcare providers expressed a positive attitude toward the FRIQuest as an instrument for identifying parents experiencing financial strain. Nevertheless, some of them questioned whether they were the most appropriate person to address patients’ financial vulnerability, partly because of limited time during consultations, and partly because of their role as specialised providers who primarily focus on their medical domain. They suggested that nurses or physician assistants might be better positioned to take on this responsibility, as these providers often hold a broader perspective on patients’ overall wellbeing. When asked whether discussing financial issues was considered part of the healthcare provider’s role, one respondent remarked:

“I’m a bit hesitant about that, because, look, you naturally want something to take on a much more meaningful meaningwhen discussed by the doctor, and perhaps you should just keep it a bit more within the realm of normal. For example, the same doctor’s assistant might already see that, and then they’ll help you, like, ‘Oh, I see this and that, and I know that too’.” Healthcare provider 1

#### Comfort while completing FRIQuest

Within the survey and the interviews, it emerged that parents felt surprised when asked about their financial vulnerability through FRIQuest. Interviewees who had been attending Erasmus MC for a longer time noted that such questions were new. To the question of how comfortable parents felt while completing FRIQuest, 22 survey respondents (37.9%) answered they felt very comfortable, 5 (8.6%) felt slightly comfortable, 23 (39.7%) felt neutral, 7 (12.1%) felt slightly uncomfortable, and one parent (1.7%) felt very uncomfortable. Some respondents additionally shared they find financial matters a private concern (*n* = 2) or that they felt comfortable but can imagine it feels different for those who experience financial vulnerability (*n* = 1). Feelings regarding comfort were further explored in the interviews, and varied:

“Well, I can imagine that [if you have financial vulnerability] it [FRIQuest] might feel like an invasion of your personal life [...] of course there’s also a sense of shame around financial vulnerability, so I can imagine that the feeling about it might be somewhat less positive.[...] I have nothing to hide, so in that sense it doesn’t bother me.” Parent 3 (no financial vulnerability)

“Because it [FRIQuest] wasn’t tiring; that nagging feeling didn’t come into it. [...]It didn’t come across as pushy. It felt very, well, safe to me.” Parent 4 (currently experiencing financial vulnerability)

One participant further explained that they found the FRIQuest somewhat confronting and needed a moment to reflect, as it can trigger one’s past with financial vulnerability.

#### Digital questionnaire as a method

Participants were positive towards the use of a digital questionnaire to address financial vulnerability. Participants highlighted the optional nature of completing the FRIQuest, giving parents the option to disclose financial vulnerability to their healthcare provider. Some participants also appreciated being able to complete the FRIQuest at home, noting that this felt less intimidating than being asked similar questions during a consultation:

“I think that if she [healthcare provider] were to ask me personally [about financial matters], or something like that, I wouldn’t be completely open and honest. And I do think that with matters like these, you should be able to be open and honest.” Parent 2 (previously experienced financial vulnerability)

Another advantage was that the FRIQuest could be completed without the child being present, as not all parents feel comfortable discussing financial matters in front of them. However, because the FRIQuest results are intended to be discussed during the consultation, where children may be present, this could still pose challenges. One parent therefore suggested that offering a telephone consultation specifically to discuss the FRIQuest could be a helpful alternative.

Disadvantages of using (digital) questionnaires also came forward in the evaluation. First, not everyone has the digital skills to complete online questionnaires. Second, the FRIQuest added to the already considerable questionnaire burden experienced by parents. Third, not all parents received follow-up on their responses from their child’s healthcare provider, reinforcing the perception that completing questionnaires can feel futile:

“Because we also just had to fill in a lot of other questionnaires, because then [FRIQuest] came of course as [part of] a bundle, and then I did think, well, I spent quite a long time on it, I have to say. And when you then come to the outpatient clinic and no one actually asks about it, yes, I did find that a bit of a waste of my time.” Parent 1 (no financial vulnerability)

#### Ease of use

In response to the question regarding the ease of completing FRIQuest, most respondents (*n* = 52, 88.1%) indicated that they found it straightforward. Interview participants described the questions as clear, the response options adequate, and the completion time manageable:

“I’ve completed in plenty of lists about financial matters. But at a certain point you think, well, forget it, I really don’t feel like filling in those stupid questions again, and then you just start clicking things without really thinking. But the focus to fill it [FRIQuest] remained there.” Parent 4 (currently experiencing financial vulnerability)

One participant described some difficulty in answering because, although she was not experiencing financial vulnerability at the time of completion, she was familiar with them and therefore was unsure what to answer.

Because the FRIQuest was integrated into the existing VBHC logistical infrastructure, its ease of use was high for healthcare providers already familiar with the VBHC infrastructure. For healthcare providers who were new to VBHC, the kick-off meeting held prior to implementation of FRIQuest informed providers how to use the VBHC dashboard. Nevertheless, to increase the ease of use, healthcare providers mentioned preferring an additional notification when parents disclose financial vulnerability, rather than requiring active retrieval from the dashboard. Additionally, healthcare providers mentioned that, in case of disclosed financial vulnerability, an automatic referral system to support services would be favourable, rather than having to initiate follow-up themselves.

## Discussion

### General findings

This study found that the FRIQuest is a generally acceptable, useful, and user-friendly instrument for supporting paediatric healthcare providers in identifying and facilitating discussions about financial vulnerability with parents. Among parents who completed the questionnaire, 22% reported financial concerns, material hardship, or both, suggesting that the digital questionnaire may help healthcare providers in identifying families with financial vulnerability when embedded within a VBHC infrastructure.

Overall, parents considered it appropriate for their child’s healthcare provider to ask about financial matters using FRIQuest, but rather as part of a general inquiry into how a family is doing than as a separate stand-alone question. Healthcare providers reported that the questionnaire was useful for identifying families in need and was easy to use in clinical practice. Still several barriers were identified. Although parents found FRIQuest easy to complete, some reported difficulties accessing the questionnaire through the digital patient portal. In addition, while healthcare providers valued FRIQuest’s ability to identify financial vulnerability, some experienced challenges in their available time for preparation and discussing these concerns with parents during the consultations.

### Financial vulnerability as a multidimensional construct

Although internationally poverty is commonly defined in terms of income thresholds and eligibility criteria for social benefits, this definition is too narrow for clinical screening. Income-based measures are useful for identifying poverty at a population level, but may not fully capture the financial circumstances that influence families’ daily lives, health-related behaviours, and access to healthcare. In contrast, financial vulnerability encompasses both subjective financial concerns and objective material hardship, recognising that families may experience financial strain in different ways and to varying degrees, regardless of whether they meet formal poverty criteria. This broader perspective is consistent with recent developments in the Netherlands, where poverty measurement increasingly recognises the importance of material hardship that prevents the ability to afford essential needs and participate in society, in addition to income alone [24].

In addition, our study shows that subjective financial concerns and objective material hardship do not always coincide. Some parents reported no overall financial concerns while experiencing difficulties in affording healthy food, medical care, participation in sports, and covering fixed expenses. Others reported financial concerns without corresponding material constraints. This highlights that families can experience financial vulnerability in different ways, and each of these may be relevant to healthcare access, use, and health-promoting behaviours. Consequently, FRIQuest may identify families who could be missed by more narrowly focused screening approaches. Poverty remains the broader societal context that motivates this work, while financial vulnerability reflects the heterogeneous ways in which financial concerns and material hardship manifest at the family level.

### Feasibility and implementation considerations

Our findings suggest that a digital screening questionnaire for financial vulnerability is feasible and acceptable within a hospital setting. Implementation benefited from embedding the FRIQuest into existing structures. This not only minimised adjustments to healthcare providers’ workflow but may also have enhanced acceptability and user friendliness among patients and families, who were already familiar with the system and its use within routine care. Nevertheless, the parent response rate and healthcare provider compliance rate also highlight some implementation challenges.

Compared to Zheng et al. our response rate was lower (40.2% vs. 92.0%) [16]. This difference is likely explained by the fact that Zheng et al. administered the questionnaire in person by psychosocial staff, plausibly increasing participation. In contrast, in our study, completion of the FRIQuest relied more heavily on parent initiative and digital skills. This reliance may have reduced overall participation. In addition, it may have introduced selection bias, as barriers to completion identified in this study, including limited digital access, lower digital literacy, difficulties accessing the patient portal, and language barriers, are likely to disproportionately affect families experiencing financial vulnerability [25–27]. Consequently, the observed prevalence of financial vulnerability should be interpreted with caution, as the families most at risk may have been underrepresented among respondents. To ensure digital questionnaires are accessible for all parents, future work should focus on developing more inclusive strategies, especially for those with language barriers and lower levels of (digital) literacy. This may involve designing simplified digital interfaces, as well as providing clear explanations and support throughout the process [28]. In addition, the modest completion rate may partly reflect reluctance among some parents to disclose financial circumstances. Although most parents who completed the FRIQuest reported feeling comfortable answering the questions and considered it useful for healthcare providers to be aware of financial vulnerability, some respondents questioned the relevance of discussing financial circumstances in a hospital setting. Furthermore, some parents who did not complete the FRIQuest reported that they were unwilling to share this information with their healthcare provider. These findings suggest that, alongside reducing practical barriers to completion, greater emphasis may be needed on explaining the relationship between financial vulnerability and child health and healthcare access to increase understanding and acceptance of screening.

Moreover, this study revealed substantial variation in compliance rates and the extent to which FRIQuest responses were discussed during consultations. This may, in part, be explained by differences in providers’ prior experiences with social determinants of health (SDoH) screening, which involves consideration of non-medical factors that influence the health of patients. Evidence from a systematic scoping review indicates that providers with experience in SDoH screening report more positive attitudes, improved knowledge, and higher screening rates, whereas those with limited exposure often report insufficient knowledge and uncertainty about their ability to address SDoH with patients as barriers [29]. However, attention to SDoH is not yet structurally embedded in routine healthcare practice, and some healthcare providers in this study experienced some challenges in initiating conversations about financial vulnerability. These findings underscore the need for training and practical guidance to equip healthcare professionals with the skills and confidence to address financial vulnerability in a sensitive and patient-centred manner.

Last, identifying financial vulnerability does not in itself ensure access to adequate support. This study found that follow-up after a positive FRIQuest screening by healthcare providers was limited, reflecting a broader challenge in translating identification into action. Several factors may have contributed to this gap, including limited consultation time, competing clinical priorities, and uncertainty among healthcare providers about discussing financial vulnerability or addressing identified needs. In addition, follow-up may take different forms depending on a family’s situation and needs. For some families, discussing financial vulnerability may help healthcare providers better understand their circumstances and adapt care accordingly, while others may benefit from referral to support services. To address the gap between identification and follow-up, structured referral pathways have been developed as part of this project in collaboration with municipal social support services. Because many needs associated with financial vulnerability fall outside the healthcare domain, effective follow-up often requires coordination between healthcare and social services. Embedding access to social support within the hospital setting may therefore facilitate timely referral to appropriate assistance. Nevertheless, healthcare providers in this study expressed a need for more automatic referral options following a positive screening. Whether such strategies improve follow-up and support for families remains unclear and warrants further investigation.

Ultimately, follow-up studies are needed to assess whether the proposed strategies improve response and compliance rates. Furthermore, future research should evaluate the effectiveness of referral pathways and examine whether systematic financial vulnerability screening with the FRIQuest contributes to increased referral rates to financial support services. Longitudinal studies are also needed to explore how financial vulnerability among these families evolves over time.

### Strengths and limitations

This study has several strengths. First, by including both parents and healthcare providers and applying a mixed-methods design, it captured a broad range of perspectives on the use of FRIQuest. Furthermore, the open-ended survey question for parents provided additional depth to the quantitative findings. Conducting the study across four distinct paediatric subspecialties increased the diversity of patient populations and care contexts represented, suggesting that the findings may be relevant across a range of paediatric outpatient settings. In addition, evaluating the digital screening questionnaire as part of VBHC provided insight into factors influencing its uptake and use, further enhancing the relevance of the findings for implementation in similar paediatric healthcare settings. In addition, although the median neighbourhood socioeconomic score of the survey respondents was slightly higher than the national average (Supplementary file 6), the range of scores indicates that families from neighbourhoods spanning different socioeconomic positions were represented.

However, several limitations should be considered. First, this study focused on the implementation, perceived usefulness, acceptability, and user friendliness of FRIQuest rather than its effectiveness as a screening instrument. As its validity and reliability were not formally evaluated, no conclusions can be drawn regarding its accuracy in identifying financially vulnerable families. Future research is needed to determine whether FRIQuest can accurately identify these families. Second, because FRIQuest and the evaluation survey were administered digitally, families facing barriers related to digital access and digital literacy may have been less likely to participate. In addition, the FRIQuest was available in Dutch and English, and the evaluation survey was available in Dutch only, which may have further limited participation among non-Dutch speaking families. As these barriers may be more common among financially vulnerable families, selection bias may have occurred, resulting in an underrepresentation of the most vulnerable families. Consequently, the prevalence of financial vulnerability in the survey may have been underestimated, and the generalisability of the survey findings may be limited. Third, participation in interviews was limited to a selected group of parents who completed FRIQuest and consented to follow-up contact. Parents reporting more negative experiences seemed less likely to engage in follow-up interviews, potentially resulting in an overrepresentation of more positive views regarding the acceptability and user friendliness of FRIQuest. In addition, only one interviewed parent was currently experiencing financial vulnerability. Therefore, the parent interviews should primarily be interpreted as providing contextual understanding and supporting the interpretation of the quantitative findings, rather than as a comprehensive representation of the experiences of all families receiving the FRIQuest.

## Conclusion

This study shows that FRIQuest is a feasible, generally acceptable, and potentially useful digital questionnaire for supporting the identification and facilitating the discussion of financial vulnerability in paediatric hospital care. Most parents and healthcare providers regarded it as appropriate to address financial circumstances within clinical consultations, particularly when embedded in broader discussions of family wellbeing. By combining subjective financial vulnerability with indicators of material hardship, FRIQuest aims to address different forms of poverty that may otherwise remain unrecognised.

While the findings suggest that FRIQuest has potential as a screening tool, important challenges remain. Barriers among parents and healthcare providers, such as digital access, time constraints, and workflow integration, were evident, which may limit healthcare provider engagement and equitable participation and meaningful support for families. These findings highlight that successful implementation of financial vulnerability screening requires not only suitable questionnaires, but also inclusive implementation strategies, clear communication about the relevance of financial vulnerability to health and healthcare access, provider engagement and support, integration in clinical pathways, and embedding access to social support. Future research should examine whether systematic screening by healthcare professionals improves referral rates to financial support services and reduces socioeconomic inequalities in child health.

## Supplementary Information

Below is the link to the electronic supplementary material.

ESM 1 Supplementary Material 1 (DOCX 52.4 KB)

## Data Availability

The data supporting the findings of this study include interview data, responses to an evaluation questionnaire, and data collected using the FRIQuest in clinical practice. Aggregated results are provided in the manuscript and supplementary materials. Due to the sensitive nature of the interview data and the scope of participant consent, the interview transcripts are not publicly available.

## Abbreviations

FRIQuest: Financial Risk Questionnaire
SDOH: Social determinants of health
VBHC: Value-based health care
