# Supplementary material for: Screening for financial vulnerability in a paediatric hospital setting: a pilot study of the Financial Risk Questionnaire (FRIQuest)
Source: Eur J Pediatr. 2026 Oct 12;185(11):832. doi: 10.1007/s00431-026-07474-5 (PMC13658393; doi:10.1007/s00431-026-07474-5)
Supplement: Supplementary file 1 [file 431_2026_7474_MOESM1_ESM.docx]

**Supplementary information**

**Supplementary file 1 - the consolidated criteria for reporting qualitative research (COREQ)**
**checklist**
**Supplementary file 2 - the Financial RIsk Questionnaire (FRIQuest)**
**Supplementary file 3 - Evaluation survey of FRIQuest for parents of patients**
**Supplementary file 4 - Survey results and characteristics of respondents to survey**
**Supplementary file 5 - Interview guide for parents of patients**
**Supplementary file 6 - Interview guide for healthcare providers**
**Supplementary file 7 – Reflexivity of interviewers and coders**

**Supplementary file 1 - the consolidated criteria for reporting qualitative research (COREQ) checklist**

| **No. Item** | **Guide questions/description** | **Reported on Page #** |
| --- | --- | --- |
| **Domain 1: Research team and reﬂexivity** |  |  |
| *Personal Characteristics* |  |  |
| 1. Inter viewer/facilitator | Which author/s conducted the interview or focus group? | 7 |
| 2. Credentials | What were the researcher’s credentials? E.g. PhD, MD | Supplementary file 7 |
| 3. Occupation | What was their occupation at the time of the study? | Supplementary file 7 |
| 4. Gender | Was the researcher male or female? | Supplementary file 7 |
| 5. Experience and training | What experience or training did the researcher have? | Supplementary file 7 |
| *Relationship with participants* |  |  |
| 6. Relationship established | Was a relationship established prior to study commencement? | Supplementary file 7 |
| 7. Participant knowledge of the interviewer | What did the participants know about the researcher? e.g. personal goals, reasons for doing the research | Supplementary file 7 |
| 8. Interviewer characteristics | What characteristics were reported about the inter viewer/facilitator? e.g. Bias, assumptions, reasons and interests in the research topic | Supplementary file 7 |
| **Domain 2: study design** |  |  |
| Theoretical framework |  |  |
| 9. Methodological orientation and Theory | What methodological orientation was stated to underpin the study? e.g. grounded theory, discourse analysis, ethnography, phenomenology, content analysis | 7 |
| Participant selection |  |  |
| 10. Sampling | How were participants selected? e.g. purposive, convenience, consecutive, snowball | 6 |
| 11. Method of approach | How were participants approached? e.g. face-to-face, telephone, mail, email | 6 |
| 12. Sample size | How many participants were in the study? | 6 |
| 13. Non-participation | How many people refused to participate or dropped out? Reasons? | 6 |
| Setting |  |  |
| 14. Setting of data collection | Where was the data collected? e.g. home, clinic, workplace | 6 |
| 15. Presence of non-participants | Was anyone else present besides the participants and researchers? | 6 |
| 16. Description of sample | What are the important characteristics of the sample? e.g. demographic data, date | Supplementary file 4 |
| Data collection |  |  |
| 17. Interview guide | Were questions, prompts, guides provided by the authors? Was it pilot tested? | Supplementary file 5 and 6 |
| 18. Repeat interviews | Were repeat inter views carried out? If yes, how many? | 7 |
| 19. Audio/visual recording | Did the research use audio or visual recording to collect the data? | 7 |
| 20. Field notes | Were ﬁeld notes made during and/or after the inter view or focus group? | 7 |
| 21. Duration | What was the duration of the inter views or focus group? | 7 |
| 22. Data saturation | Was data saturation discussed? | 6 |
| 23. Transcripts returned | Were transcripts returned to participants for comment and/or correction? | 7 |
| **Domain 3: analysis and ﬁndings** |  |  |
| Data analysis |  |  |
| 24. Number of data coders | How many data coders coded the data? | 7 |
| 25. Description of the coding tree | Did authors provide a description of the coding tree? | 7 |
| 26. Derivation of themes | Were themes identiﬁed in advance or derived from the data? | 7 |
| 27. Software | What software, if applicable, was used to manage the data? | 7 |
| 28. Participant checking | Did participants provide feedback on the ﬁndings? | 7 |
| Reporting |  |  |
| 29. Quotations presented | Were participant quotations presented to illustrate the themes/ﬁndings? Was each quotation identiﬁed? e.g. participant number | 8-12 |
| 30. Data and ﬁndings consistent | Was there consistency between the data presented and the ﬁndings? | 8-12 |
| 31. Clarity of major themes | Were major themes clearly presented in the ﬁndings? | 8-12 |
| 32. Clarity of minor themes | Is there a description of diverse cases or discussion of minor themes? | 8-12 |

**Supplementary file 2 – the Financial RIsk Questionnaire (FRIQuest)**

**Questionnaire Financial matters** *(title displayed towards parents)*

Financial concerns can affect your child’s health and access to care. Conversely, having a sick child can lead to financial concerns, for example because you can work less. Therefore, it is important for your healthcare provider to know whether there are financial concerns. They can try to take this into account in the treatment and, if desired, refer you to support.

1. **Do you have concerns about financial matters?**

- Yes, I am concerned about financial matters.
- I am occasionally concerned about financial matters.
- No, I am not concerned about financial matters.

2. **Do you have enough money to cover fixed expenses?**

***Consider, for example, rent, or bills for utilities like energy, water, or internet.***

- Yes, I have enough money for fixed expenses.
- I sometimes have insufficient money for fixed expenses.
- No, I do not have enough money for fixed expenses.

3. **Do you have enough money to buy healthy food?**
   ***Consider, for example, fruit and vegetables.***

- Yes, I have enough money to eat healthily.
- I sometimes have insufficient money to eat healthily.
- No, I do not have enough money to eat healthily.

4. **Do you have enough money for sports or exercise for your child(ren)?**

- Yes, I have enough money for sports or exercise.
- I sometimes have insufficient money for sports or exercise.
- No, I do not have enough money for sports or exercise.

5. **Do you have enough money to pay for medical care?**

***Consider things like the deductible or costs for transportation to the hospital.***

- Yes, I have enough money for medical care.
- I sometimes have insufficient money for medical care.
- No, I do not have enough money for medical care.

6. **Do you want (more) help with your financial concerns?**

- Yes, I want (more) help.
- No, I already receive sufficient help with my financial concerns.
- No, I do not want help.

7. **Is there anything else you would like to share?**

*Open-ended question*

**Supplementary file 3 – Evaluation survey of FRIQuest for parents of patients**

You recently had an appointment with your child’s healthcare provider in Erasmus MC. Before this visit, you were asked to complete several questionnaires digitally. These included some questions about financial matters. You may or may not have completed these questionnaires. Your healthcare provider may or may not have discussed them with you.

We would like to ask you a few questions about your experience with the questions about financial matters. Completing this questionnaire takes 5–10 minutes and is anonymous. This means that no one knows which answers are yours. If you do not want to answer a question, then you do not have to.

For questions you can contact the department of Quality and Patient care, via [e-mail address].

**1. Did you complete the questions about financial matters?**

- Yes, I completed them *(followed by question 3)*
- No, I did not complete them *(followed by question 2)*
- I do not know whether I completed them *(followed by question 11)*

**2. Why did you not complete the questions about financial matters? *You may select multiple answers.***

- I did not have a computer or laptop
- I did not have DigiD
- I did not know I had to complete the questionnaire
- I noticed too late that I had received the questionnaire
- I did not have time to complete the questionnaire
- I did not know how to complete the questionnaire
- I did not understand the questions in the questionnaire
- I felt the questions were inappropriate
- I felt the questions did not apply to my situation
- I did not want to share my answers with my healthcare provider
- Other, namely …

*Followed by question 11*

*Questions 3-10 were only shown when the answer to question 1 was “Yes, I completed them”.*

**3. How comfortable did you feel completing the questions about financial matters?**

- I felt very uncomfortable
- I felt somewhat uncomfortable
- I felt neutral: neither uncomfortable nor comfortable
- I felt somewhat comfortable
- I felt very comfortable

**4. Did you find it difficult to complete the questions about financial matters?**

- Yes, I found it difficult
- I did not find it difficult, but not easy either
- No, I found it easy

**5. Do you find it useful that your healthcare provider knows, based on the questions about financial matters, whether financial concerns are present for you?**

- Yes, I find it useful that my healthcare provider knows this
- I find it neither useful nor useless that my healthcare provider knows this
- No, I find it useless that my healthcare provider knows this

**6. Did you indicate in the questions about financial matters that you have (some) concerns about finances? *Think about paying bills, healthy food, or sports for your child.***

- Yes, I indicated this
- No, I did not indicate this
- I do not remember
- I do not want to say

**7. Did you answer the questions about financial matters truthfully?**

- Yes, I answered everything truthfully
- No, I did not answer everything truthfully
- I do not remember
- I do not want to say

*Question 8 is only shown if question 6 was answered with “Yes, I indicated this”.*

**8. Did your healthcare provider discuss the questions about financial matters with you?**

- Yes, my healthcare provider discussed them with me (followed by question 9)
- I raised my financial concerns myself towards my healthcare provider (followed by question 9)
- My healthcare provider briefly mentioned the questions but did not discuss them *(followed by question 11)*
- No, my healthcare provider did not discuss them *(followed by question 11)*
- I do not remember *(followed by question 11)*
- Other, namely … *(followed by question 11)*

*Questions 9 and 10 are only shown if question 8 was answered with “Yes,* my healthcare provider discussed them with me*” or “I raised my financial concerns myself towards my healthcare provider ”.*

**9. What did your healthcare provider do or mention to help you with financial concerns? *You may select multiple answers.***

- Pointed me to organizations I could contact myself
- Referred me to support within the hospital (e.g. medical social work or the patient service center)
- Referred me to support outside the hospital (e.g. community team or debt assistance)
- Adjusted my treatment plan (e.g. fewer hospital visits or different medication)
- Did nothing to help with my financial concerns
- Other, namely …

**10. Are you satisfied with the conversation with your healthcare provider about financial matters?**

- I am very dissatisfied
- I am somewhat dissatisfied
- I am neither dissatisfied nor satisfied
- I am somewhat satisfied
- I am very satisfied

**11. Would you like to add anything about this topic?**
*Open-ended question*

The following questions help us better understand your answers.

**12. What is your gender?**

- Male
- Female
- Other

**13. What is your age?**
*Open-ended question*

**14. Which medical department of the Erasmus MC did you visit?**
*Open-ended question*

**15. What is the age of your child for whom you had an appointment at the Erasmus MC?**
*Open-ended question*

**16. What are the first four digits of your postal code?**
*Open-ended question*

We would like to improve our services at Erasmus MC. For this purpose, we would like to use your questionnaire responses anonymously for research. Anonymous means that no one knows which answers are yours.

We will store your responses for 10 years for research purposes. The data are stored on a secure hospital computer and can only be accessed by the researchers, who have signed a confidentiality agreement.

Participation is voluntary. If you do not wish to participate, there will be no consequences for you. The results will be published in a scientific journal.

For questions, you can contact Ashley Smit, researcher at Erasmus MC, via [telephone number].

**17. Do you consent to the use of your answers for research purposes?**

- Yes, I give consent
- No, I do not give consent

We would like to learn how we can better support our patients. May we contact you for follow-up research? If you participate, you will receive compensation.

**18. May we contact you for follow-up research?**

- Yes, they may contact me for follow-up research
- No, they may not contact me for follow-up research

Question 19 is only shown when question 18 is answered with “Yes, they may contact me for follow-up research”.

**19. What is your phone number?**
*Open-ended question*

**Supplementary file 4 – Evaluation survey results and characteristics of survey respondents**

| **Table 1. Evaluation survey results and characteristics of survey respondents (N=112)** | | |
| --- | --- | --- |
| **Survey question (number of respondents who should have answered the question)** | **Answer options** | **n, (%)** |
| 1. Did you complete the questions about financial matters? (n=112) | Yes, I completed them | 66 (58.9) |
|  | No, I did not complete them | 31 (27.7) |
|  | I do not know whether I completed them | 15 (13.4) |
| 2. Why did you not complete the questions about financial matters?^a^ (n=31) | I did not have a computer or laptop | 2 (6.5) |
|  | I did not have DigiD^b^ | 7 (22.6) |
|  | I did not know I had to complete the questionnaire | 10 (32.3) |
|  | I noticed too late that I had received the questionnaire | 6 (19.4) |
|  | I did not have time to complete the questionnaire | 1 (3.2) |
|  | I did not know how to complete the questionnaire | 2 (6.5) |
|  | I did not understand the questions in the questionnaire | 0 |
|  | I felt the questions were inappropriate | 0 |
|  | I felt the questions did not apply to my situation | 3 (9.7) |
|  | I did not want to share my answers with my healthcare provider | 2 (6.5) |
|  | Other  Parents could not find the questionnaire  Questionnaire display problems | 3 (9.7)  1 (3.2) |
|  | *Missing* | *0* |
| 3. How comfortable did you feel completing the questions about financial matters? (n=66) | I felt very uncomfortable | 1 (1.7) |
|  | I felt somewhat uncomfortable | 7 (12.1) |
|  | I felt neutral: neither uncomfortable nor comfortable | 23 (39.7) |
|  | I felt somewhat comfortable | 5 (8.6) |
|  | I felt very comfortable | 22 (37.9) |
|  | *Missing* | *8* |
| 4. Did you find it difficult to complete the questions about financial matters? (n=66) | Yes, I found it difficult | 0 (0) |
|  | I did not find it difficult, but not easy either | 7 (11.9) |
|  | No, I found it easy | 52 (88.1) |
|  | *Missing* | *7* |
| 5. Do you find it useful that your healthcare provider knows, based on the questions about financial matters, whether financial concerns are present for you? (n=66) | No, I find it useless that my healthcare provider knows this | 11 (21.2) |
|  | I find it neither useful nor useless that my healthcare provider knows this | 13 (25.0) |
|  | Yes, I find it useful that my healthcare provider knows this | 28 (53.8) |
|  | *Missing* | *14* |
| 6. Did you indicate in the questions about financial matters that you have (some) concerns about finances? *(n=66)* | Yes, I indicated this | 6 (10.9) |
|  | No, I did not indicate this | 46 (83.6) |
|  | I do not remember | 3 (5.5) |
|  | I do not want to say | 0 |
|  | *Missing* | *11* |
| 7. Did you answer the questions about financial matters truthfully? (n=66) | Yes, I answered everything truthfully | 59 (100) |
|  | No, I did not answer everything truthfully | 0 |
|  | I do not remember | 0 |
|  | I do not want to say | 0 |
|  | *Missing* | *7* |
| 8. Did your healthcare provider discuss the questions about financial matters with you?^c^ (n=66) | Yes, my healthcare provider discussed them with me | 1 (1.9) |
|  | I raised my financial concerns myself towards my healthcare provider | 1 (1.9) |
|  | My healthcare provider briefly mentioned the questions but did not discuss them | 0 |
|  | No, my healthcare provider did not discuss them | 51 (94.4) |
|  | I do not remember | 0 |
|  | Other  No, because I filled in the questionnaire after my appointment | 1 (1.9) |
|  | *Missing* | *12* |
| 9. What did your healthcare provider do or mention to help you with financial concerns?**^a^** (n=2) | Pointed me to organizations I could contact myself | 1 (50) |
|  | Referred me to support within the hospital | 0 |
|  | Referred me to support outside the hospital | 0 |
|  | Adjusted my treatment plan | 0 |
|  | Did nothing to help with my financial concerns | 1 (50) |
| 10. Are you satisfied with the conversation with your healthcare provider about financial matters? (n=2) | I am very dissatisfied | 0 |
|  | I am somewhat dissatisfied | 0 |
|  | I am neither dissatisfied nor satisfied | 0 |
|  | I am somewhat satisfied | 0 |
|  | I am very satisfied | 2 (100) |
| **Survey question (number of respondents who answered the question)** | **Thematic categorization of answers** | **n, (%)** |
| 11. Would you like to add anything about this topic? (n=15) | Expressing the importance of attention for financial matters | 5 (33.3) |
|  | Confirm the absence of financial concerns or material hardship | 5 (33.3) |
|  | Expressing surprise or displeasure about receiving FRIQuest | 4 (26.7) |
|  | Comment or question about the goal of the FRIQuest | 3 (20.0) |
|  | Expressing understanding people's reluctance to discuss financial concerns | 2 (13.3) |
|  | Suggestions to include a question on relevance in FRIQuest | 2 (13.3) |
|  | Comment on high travel and/or healthcare expenses | 2 (13.3) |
|  | Suggestion to provide an overview of benefits and allowances you may be entitled to | 1 (6.7) |
| **^a^** Multiple answers possible  ^b^ DigiD: digital identification necessary to access the patient portal  ^c^ Note that healthcare providers were not explicitly instructed to discuss the FRIQuest unless it identified previously  unrecognized poverty | | |

| **Table 2. Characteristics of survey respondents (N=112)** | |
| --- | --- |
| **Characteristic** | **Number (%) or Median (IQR)^a^** |
| **Sex**  Male  Female  *Missing* | 23 (22.3%) 81 (77.7%) *8* |
| **Age (in years)**  Range  *Missing* | 40 (36-45)  28 - 62  *1* |
| **SES-WOA score^b^**  Range  *Missing* | 0.1415 (-0.0380 – 0.2473)  -0.5510 – 0.5760  *8* |
| ^a^ IQR: Inter-quartile range, exclusive method  ^b^ SES-WOA refers to a standardized indicator developed by the Central Bureau of Statistics Netherlands, that summarizes socio‑economic position of neighborhoods based on household wealth, educational attainment, and recent employment history; 0 is the average and higher scores indicate a more advantaged socio‑economic position. [[cbs.nl]](https://www.cbs.nl/nl-nl/faq/infoservice/hoe-interpreteer-je-de-ses-woa-scores-en-hoe-zijn-deze-bepaald-) | |

**Supplementary file 5 – Interview guide for parents of patients**

You recently completed a questionnaire evaluating the financial matters questionnaire. During this interview, we would like to further explore your answers to gain better insight into whether the financial matters questionnaire helps to identify and support patients with financial concerns.

**1. Can you tell us which department(s) at Erasmus MC your child is a patient of, and how often you have been there?**

**2. You received a questionnaire about financial concerns. What did you think of this questionnaire?**

- What feeling did you have while completing it?
- How easy or difficult did you find it to complete the questionnaire?
- Was there any question you found inappropriate?
- Is there a question you felt was missing from the questionnaire?

**3. How do you feel about your healthcare provider asking whether you have financial concerns?**

- Do you find this appropriate?
- Why do you think your healthcare provider asks about financial concerns?

**4. How do you feel about [medical department] using a questionnaire to ask about financial concerns?**

- Is a questionnaire a good way to ask about this?
- How would you feel if these questions were asked during your appointment by the healthcare provider?
- What do you prefer: a questionnaire, or the healthcare provider asking these questions? Or do you see another way to ask about financial concerns?
- What do you think about the questionnaire being digital? Would you prefer to complete it on paper?
- What do you think about receiving this questionnaire before your appointment? Is this the right moment?

**5. Depending on the answers given in the evaluation questionnaire, ask about responses.** For example: I see that you indicated that you felt uncomfortable answering the questions on financial matters. Can you elaborate on this?

**6. Can you tell us about your appointment with your healthcare provider? Did your healthcare provider ask about financial concerns?**

If yes:

- What did the healthcare provider ask you?
- How did this question make you feel?
- Was the “financial matters” questionnaire mentioned?
- What did you discuss with the doctor about financial matters?

If no:

- What do you think about your healthcare provider not asking about this?
- Was the “financial matters” questionnaire mentioned?
- Did the doctor discuss other costs with you, such as transportation costs, food costs, or sports costs?

**7. Are you familiar with the support available at Erasmus MC for people with financial concerns?** If “no”, mention Medical Social Work and the financial support team of the Patient Service Center.

- Were you referred by your healthcare provider for help with financial concerns?
- Where were you referred to?
- What do you think about the fact that Erasmus MC offers support for people with financial concerns?
- What do you think is important to ensure that people are able and willing to use this support?

**8. If financial concerns were present: Did your healthcare provider say or do anything after the first conversation about your financial concerns?**

- Are you still in treatment with your healthcare provider?
- When is/was your second appointment?

**9. What is currently missing in the hospital that could better support patients with financial concerns?**

**Supplementary file 6 – Interview guide for healthcare providers**

We want to use this interview to find out whether the FRIQuest has made a difference in how financial concerns among patients are identified and discussed. We are particularly interested in your personal experience: how was this before the use of the questionnaire, and how is it now? At the end of the interview, we will share the results of the FRIQuest to date with you.

**1. Can you reflect on how you experienced identifying and discussing financial concerns before the questionnaire, and what is different now that you are working with the questionnaire?**

**2. Has the questionnaire made it easier or more difficult to discuss financial concerns? And how do you notice this?**

**3. Are there situations in which you think: this is something I might not have noticed or discussed without the questionnaire?**

**4. To what extent do you know where to refer parents of patients with financial concerns if they want additional support?**

**5. What is currently missing that could help you better identify and support patients with financial concerns?**

**Supplementary file 7 – Reflexivity of interviewers and coders**

AJPS (MSc, female), LGS (MSc, female) and BQA (MSc, female) had followed courses in qualitative research and had experience in qualitative methods prior to conducting the interviews. At the time of the study, AJPS was a PhD student in the field of pediatrics and LGS and BQA were occupied as advisors for appropriate care at the directorate Quality and Patientcare. The interviewers had an established relationship with participating healthcare providers, but not with participating parents before the interviews. Recruitment through their healthcare organization likely fostered a sense of trust and legitimacy among participants. Participants were informed about the interviewers’ academic role and the purpose of the study before the interview commenced. All interviewers reflected on their positionality and potential influence on data collection throughout the research process. All interviewers were motivated by an interest in improving health disparities, which may have shaped their expectations about the importance of certain themes. They acknowledged these assumptions during the research process and engaged in reflexive discussions to minimize bias in data collection and coding.
